# ESET methylates UBF at K232/254 and regulates nucleolar heterochromatin plasticity and rDNA transcription

**DOI:** 10.1093/nar/gkt1041

**Published:** 2013-11-14

**Authors:** Yu Jin Hwang, Dohyun Han, Ki Yoon Kim, Sun-Joon Min, Neil W. Kowall, Liu Yang, Junghee Lee, Youngsoo Kim, Hoon Ryu

**Affiliations:** ^1^Department of Biomedical Sciences, World Class University Neurocytomics Group, Seoul National University College of Medicine, Seoul 110-799, South Korea, ^2^Medical Engineering, Seoul National University College of Medicine, Seoul 110-799, South Korea, ^3^Center for Neuro-Medicine, Brain Science Institute, Korea Institute of Science and Technology (KIST), Seoul 136-791, South Korea, ^4^VA Boston Healthcare System, Boston, MA 02130, USA, ^5^Boston University Alzheimer’s Disease Center and Department of Neurology, Boston University School of Medicine, Boston, MA 02118, USA, ^6^Department of Orthopedics and Division of Hematology, University of Washington School of Medicine, Seattle, WA 98195, USA and ^7^Medical Research Service, VA Puget Sound Health Care System, Seattle, WA 98108, USA

## Abstract

The remodeling of chromatin in the nucleolus is important for the control of ribosomal DNA (rDNA) transcription and ribosome biogenesis. Herein, we found that upstream binding factor (UBF) interacts with ESET, a histone H3K9 methyltransferase and is trimethylated at Lys (K) 232/254 by ESET. UBF trimethylation leads to nucleolar chromatin condensation and decreased rDNA transcriptional activity. UBF mutations at K232/254A and K232/254R restored rDNA transcriptional activity in response to ESET. Both ESET-ΔSET mutant and knockdown of ESET by short hairpin RNA reduced trimethylation of UBF and resulted in the restoration of rDNA transcription. Atomic force microscopy confirmed that UBF trimethylated by ESET modulates the plasticity of nucleolar chromatin. We further demonstrated that UBF trimethylation at K232/254 by ESET deregulates rDNA transcription in a cell model of Huntington’s disease. Together, our findings show that a novel epigenetic modification of UBF is linked to impaired rDNA transcription and nucleolar chromatin remodeling, which may play key roles in the pathogenesis of neurodegeneration.

## INTRODUCTION

The nucleolus is a subnuclear component of the transcription machinery of ribosomal genes where the ribosomal DNA (rDNA) is organized as tandem repeats and is transcribed into 47S precursor ribosomal RNA (rRNA) (1). The nucleolar chromatin structure modulates the transcription of rDNA by landscaping histones and a specialized transcription complex, consisting of RNA polymerase I (RNA Pol I) and other co-regulatory factors (1,2). The transcription factor upstream binding factor (UBF) constitutes active nucleolar organizer regions (NORs) and maintain the transcriptional activity of rDNA by activating promoter-specific RNA Pol I and increasing local concentration of RNA Pol I and transcription initiation factor selectivity factor 1 (2–4). UBF is a member of the high mobility group (HMG) protein family and contains six HMG box DNA-binding motifs. Two conserved isoforms, UBF1 and UBF2, are found in many types of cells (5–7)*.* UBF1, a 97 kDa polypeptide, serves a structural role by binding to DNA across the entire rDNA repeat and regulates the transcription of rDNA by inducing remodeling of ribosomal gene chromatin (8–10). UBF2 is derived from the alternative splicing of a single transcript and UBF1 and UBF2 form hetero- and homodimers (7). As the transcriptional activity of UBF1is greater than UBF2, UBF1 targeting to regions of heterochromatin is sufficient to induce large-scale chromatin decondensation in the nucleus (5,8).

Post-translational modifications such as acetylation and phosphorylation of UBF control the transcription of rDNA (11,12). cAMP response element-binding protein (CREB)-binding protein (CBP), a histone acetyltransferase (HAT) and transcriptional coactivator, contributes to UBF-mediated transcription in the nucleolus. In addition, the transcription of rDNA is regulated by the influence of two opposing processes, namely, UBF acetylation by CBP and deacetylation by histone deacetylase (HDAC) (13). In this context, CBP-dependent acetylation of UBF is linked to the transcription activation of rDNA. Interestingly, mutated proteins such as huntingtin (Htt) and atrophin with polyglutamine repeats interact with CBP molecule and block the intrinsic HAT activity of CBP (14,15). These specific interactions suggest a model in which mtHtt is closely linked to transcriptional signaling cascades associated with a number of pathophysiological mechanisms in Huntington’s disease (HD) (15–18). We recently found that CBP interacts with UBF1, specifically acetylates UBF1 at Lys (K) 352 and modulates UBF1-mediated rDNA transcription (19). Furthermore, UBF1 acetylation is reduced and subsequently UBF1-mediated rDNA transcriptional activity is impaired in cellular and animal model of HD (19). Thus, one plausible mechanism by which mutant Htt contributes to neurodegeneration may be through transcriptional deregulation and chromatin remodeling (20).

Chromatin remodeling modulates the transcription of genes through the opposing actions of histone acetylation and methylation of N-terminal lysine residues. Histone H3K9 methylation decreases transcriptional activity, whereas acetylation improves transcription (21,22). Decreased acetylation and increased methylation of histones has been found in HD mice and we have recently found that alterations of ets-related gene (ERG)-associated protein with SET domain (ESET/SETDB1) expression and H3K9me3 level are correlated with transcriptional dysfunction and neurodegeneration in HD (23). ESET is a novel histone H3K9 methyltransferase that contains both tudor and methyl-CpG binding domains that converge transcription and RNA processing factors as well as acting as a signature motif for proteins regulating methylated DNA silencing (24). ESET is involved in neuronal dysfunction through its histone methyltransferase activity and the epigenetic silencing of neuronal genes (23). To date, however, the mechanisms of nucleolar heterochromatin landscaping and rDNA transcription by ESET have not been fully investigated (25).

Given this paradigm, the aim of this study is to determine if ESET leads to the epigenetic modification of rDNA and chromatin remodeling in a neurodegenerative condition of HD. We have determined that ESET physically interacts with UBF and specifically trimethylates UBF at two lysine (K) residues (K232 and 254). The hypermethylated UBF alters the chromatin structure of nucleolus and in turn, cause transcriptional dysfunction rDNA in an ESET-dependent manner. Our results show that the status of UBF methylation by ESET involves in the plasticity of nucleolar heterochromatin and rDNA transcription.

## MATERIALS AND METHODS

### Plasmid constructs

pIRES-Luc and human rRNA-luciferase vector (pHrD-IRES-Luc) was generously provided by Dr Samson T. Jacob (Ohio State University) (26). Human UBF construct was generated by polymerase chain reaction (PCR)-based subcloning into pCMV-Flag 2A vector (Stratagene) or pGEX6T (19). UBF methylation mutants (K232A, K254A, K232/254A, K232R, K254R and K232/254R) were generated from pCMV-Flag-UBF using site-directed mutagenesis kit (TOYOBO) (Supplementary Table S1). pCMV-WT-ESET and pCMV-ESET ΔSET were generously provided by Dr David C. Schultz (27). Myc epitope-tagged expression plasmid pCS2-MT-ESET and deletion constructs were generated as previously described (24).

### *In vitro* protein methylation assay

*In vitro* methylation assay was performed using a method previously described with slight modifications (28). glutathione s-transferase (GST)–ESET and GST–UBF HMG1-6 proteins were produced in *Escherichia coli* and purified with glutathione-Sepharose 4B beads. *In vitro* methylation reactions were incubated in 1x HMT buffer (50 mM Tris–HCl, pH 8.0, 20 mM KCl, 10 mM MgCl_2_, 10 mM dithiothreitol (DTT), 250 mM sucrose) containing 10 mM *S*-[methyl] adenosylmethionine (methyl donor) (Sigma Aldrich) at 37°C for 1 h. Then, samples were washed three times with PreScission Protease cleavage buffer (50 mM Tris–HCl, pH8.0, 150 mM NaCl, 1 mM ethylenediaminetetraacetic acid (EDTA), 1 mM DTT, 0.01% NP40). In order to cut GST out from the fusion proteins, samples were incubated in cleavage solution (4% PreScission Protease stock, GE Healthcare) at 4°C for 24 h and the supernatants (GST-free proteins) were collected for liquid chromatography- mass spectrometry and liquid chromatography/tandem mass spectrometry (LC-MS/MS) analysis.

### Cell culture and transfection assays

ST*Hdh^Q7/7^* (Q7) (wild-type) and ST*Hdh^Q111/111^* (Q111) (HD knock-in striatal cell line expresses mutant huntingtin at endogenous level), were generously provided by Dr Marcy MacDonald (Harvard Medical School) (29). For transfection assays, 2.5 × 10^5^ cells were plated onto 48-well cell culture plates 24 h before transfection. LipofectamineTM 2000 reagent (Invitrogen) has been used as a transfection reagent and transfections were performed according to the manufacturer’s protocol. The luciferase activity was measured and normalized to protein concentration. The Dual Luciferase Assay kit (Promega) was used to determine the difference of internal transcriptional activity between Q7 and Q111 cells. The luciferase assay data represent the mean ± SEM (standard error of the mean) of three independent (separate) experiments.

### Chromatin immunoprecipitation

Chromatin immunoprecipitation (ChIP) for UBF binding to rDNA was performed using a CHIP assay kit (Santa Cruz Biotechnology) as described previously (30,31). Q7 and Q111 cells were crosslinked with 1% formaldehyde for 10 min at room temperature. The lysates were sonicated six times, each time for 30 s using Bioruptor (Diogenode). After centrifugation, the supernatant was diluted in ChIP dilution buffer, and then incubated overnight at 4°C with anti-UBF antibody (H-300, Santa Cruz Biotechnology). The elution and quantification of DNA was performed as previously described (30,31). The sequences used for the quantitative real-time- PCR (qRT–PCR) primers of UBF occupancy to rDNA are shown in the Supplementary Table S2.

### qRT–PCR

Total RNA was isolated from cells using a commercial extraction system (Macherey–Nagel). A total of 1 μg of RNA has been used for complementary DNA (cDNA) preparation with First Strands cDNA Synthesis Kit (TOYOBO) according to manufacturer’s protocols. cDNA from each sample was amplified by qRT–PCR using Synergy Brands, Inc. (SYBR) Green Supermix (TOYOBO). qRT–PCR cycling conditions were as follows: denaturation for 3 min at 95°C, then 40 cycles of amplification for 15 s at 95°C, 15 s at 60°C, 20 s at 70°C, and followed by 30 s at 72°C. RNA quantities were normalized using glycerolaldehyde-3-phosphate dehydrogenase (GAPDH) messenger RNA (mRNA) as a reference (30). The sequences used for the qRT–PCR primers are shown in Supplementary Table S3.

### In-gel trypsin digestion

In-gel trypsin digestion was performed as previously reported, with some minor modifications (32,33). To destain coomassie blue, excised gel pieces were incubated in 200 mM ammonium bicarbonate/50% v/v acetonitrile and then rinsed several times with 150 μl of distilled water. They were then dehydrated in 100% acetonitrile (ACN) until they turned opaque white and rehydrated with 100 mM ammonium bicarbonate until transparent. This dehydration and rehydration process was repeated about three-to-four times, and was followed by a single dehydration in 100% ACN. The gel pieces were then dried in a speed Vac. Reduction was performed at 56°C for 60 min in reduction solution (10 mM DTT and 100 mM ammonium bicarbonate). Subsequently, alkylation of cysteines was performed at room temperature for 30 min in alkylation buffer [50 mM iodoacetamide in 100 mM ammonium bicarbonate] to improve the recovery of cysteine-containing peptides and avoid disulfide bond formation and side chain modification. The gel pieces were then dried in a speed Vac and rehydrated at 47°C for 45 min in digestion buffer containing sequencing grade modified trypsin (Promega) in 50 mM ammonium bicarbonate at a concentration of 0.01 mg/ml (Promega). Excess supernatant was then removed and gel pieces were soaked in 30 μl of 50 mM ammonium bicarbonate (NH_4_HCO_3_) overnight (16 h) at 37°C. The solutions, which then contained cleaved peptides were moved to new tubes.

### LC-MS/MS analysis and data processing

Peptide samples were analyzed by EASY nano-LC (Proxeon) interfaced with a LTQ velos ion trap mass spectrometry (Thermo Electron Corporation) as previously reported (33) with some minor modifications. The nano LC separations were operated in the two column setup with a trap column (100 μm I.D × 3 cm) and an analytical column (75 μm I.D. × 15 cm) that was packed in-house with C18 resin (Magic C18-AQ 100 Å, 5 μm particles). Peptide mixtures were dissolved in 50 μl of Solution A [H_2_O/acetonitrile/Formic acid, 95:5:0.1, v/v/v (%)]. For each analysis, 10 μl of samples was loaded onto the trap column at 5 nl/min. Peptides were separated with an exponential gradient of 0–40% solution B [H_2_O/acetonitrile/Formic acid, 5:95:0.1, v/v/v (%)] over 90 min at a constant flow rate of 300 nl/min. The gradient was then ramped to 90% solution B for 15 min and back to 0% solution B for 5 min.

The mass spectrometer was operated in data-dependent mode, automatically switching between MS and MS/MS acquisition for the 10 most abundant ion peaks per MS spectrum (TOP 10). Full-scan MS spectra (m/z 300–2000) were acquired in the profile mode. An ion spray voltage was set as 2.0 kV in positive ion mode and the temperature of heated capillary was 320°C. All colloision-induced dissociation (CID) MS/MS spectra were acquired using the following parameters: normalized collision energy, 35%; ion selection threshold, 500 counts; activation Q, 0.25 and activation time, 30 ms. Dynamic exclusion was performed with repeat count of 1, 45 s repeat duration, exclusion list size of 500, exclusion duration of 60 s and ±1.5 m/z exclusion mass width.

The MS data analysis was performed using SEQUEST on Sorcerer 2 platform (Sage-N Research). All MS/MS spectra were searched against the International Protein Index human v3.74 database. The search included cysteine carbamidomethylation as fixed modification and oxidation of methionine and trimethylation of lysine and arginine as variable modification. The database search parameters were: full enzyme digest using trypsin (After KR/-) with up to three missed cleavages; a precursor ion mass tolerance of 2 Da; a fragment ion mass tolerance of 1 Da. Trimethylated peptides identified in SEQUEST were filtered and validated using Trans-Proteome Pipeline version 4.3 and Scaffold 3 software (Proteome Software Inc.). To quantify relative abundance of trimethylated peptides, we verify trimethylated peptides by manual inspection of extracted ion chromatograms (XICs) using Qual browser of Xcalibur software version 2.1 (Thermo Fisher Scientific*)*. XICs of two trimethylated peptides (DSLGK#QWSQLSDK, m/z = 767.49 and K#EYEEIMR, m/z = 569.94) were generated using a mass window of 10 parts per million (ppm) around the exact mass at RT of each peptide.

### Confocal microscopy and 3D construction of image

Indirect labeling methods were used to determine the UBF (H-300, Santa Cruz Biotechnology) (1:200), ESET (H-300, Santa Cruz Biotechnology) (1:200), Myc (9E10, Santa Cruz Biotechnology) (1:200) and Trimethyl-Histone H3 (Lys9) (Upstate Biotechnology) (1:1000). The procedures were performed as previously described (23,34). Images were analyzed using OLYMPUS FluoView FV10i confocal laser scanning microscope (Olympus). Control experiments were performed in the absence of primary antibody or in the presence of blocking peptide (23). Deconvolution and 3D construction of confocal images was performed using the AQI-X-COMBO-CWF program (Media cybernetics Inc.). Orthoslice images were reconstructed after deconvolution of confocal images. Typically, we collected a series of more than 24 confocal layers representing fluorescence data from regions of interests to develop a consolidated image representing the details illustrated. Quantitative assessment of the image was performed using the AQI-XCOMBO-CWF program.

### Bimolecular fluorescence complementation assay

pBiFC-VC155 and pBiFC-VN155 were used in the bimolecular fluorescence complementation (BiFC) systems. UBF and ESET were subcloned into pBiFC-VC155 and pBiFC-VN155 to generate pBiFC-VC155-UBF or pBiFC-VN155-ESET vector, respectively. For the analysis of fluorescence complementation between UBF and ESET, pBiFC-VC155-UBF and pBiFC-VN155-ESET were transfected into Q7 and Q111 cells as well control VC and VN plasmids using Lipofectamine LTX2000 (Invitrogen). Forty-eight hours post-transfection, cells were fixed and stained by 4′,6-diamidino-2-phenylindole (DAPI). The BiFC signals were detected using an OLYMPUS FluoView FV10i confocal laser scanning microscope (Olympus).

### Subcellular fraction

Harvested cells were washed with phosphate buffered saline (PBS) and centrifuged at 1000 rpm for 4 min at 4°C. The cell pellet was resuspended in 500 μl of ice-cold Buffer (10 mM HEPES–KOH, pH7.9, 1.5 mM MgCl2, 10 mM KCL, 0.5 mM DTT and protease inhibitors), kept on ice for 5 min and homogenized 20 times using a tight Dounce pestle. The homogenized sample was centrifuged at 1000 rpm for 5 min at 4°C to separate nuclei and other compartments. The supernatant was retained as the cytoplasmic fraction. The pellet was resuspended in 300 μl of 0.25 M sucrose buffer (containing 10 mM MgCl2) and layered over 300 μl of 0.35 M sucrose buffer (containing 0.5 mM MgCl2) and centrifuged at 2500 rpm for 5 min at 4°C. This step resulted in a separation of cleaner nuclear fraction. Then, the nuclear fraction was resuspended in 300 μl of 0.35 M sucrose and sonicated six times with each time for 10 s using Bioruptor (Diogenode). To gain the nucleoli fraction, the sonicated sample was layered over 300 μl of 0.88 M sucrose (containing 0.5 mM MgCl2) and centrifuged at 3500 rpm for 10 min at 4°C. The pellet contained the nucleoli fraction and the supernatant was retained as the nucleoplasmic fraction. In order to obtain the nucleoli lysate, the nucleoli fraction was resuspended in radioimmunoprecipitation assay (RIPA) buffer (350 mM NaCl, 1% Triton X-100) and centrifuged at 14 000rpm for 10 min at 4°C.

### Western blot analysis

Western blot was performed as previously described (23,35,36). A total of 30 μg of protein was subjected to SDS–PAGE and blotted with anti-UBF (H-300, Santa Cruz Biotechnology), anti-ESET (H-300, Santa Cruz Biotechnology), anti-methylated lysine (tri) (AbCam, Cambridge, MA, USA), anti-methylated lysine (mono, di) (AbCam), anti-Myc (9E10, Santa Cruz Biotechnology) and anti-Flag (M2, Sigma Aldrich) antibody. The equal amount of protein loading was detected and normalized with β-actin (Santa Cruz Bitotechnology) or Histone H3 (FL136, Santa Cruz Biotechnology) on the same membrane. We developed western blot membranes with enhanced chemiluminescence (ECL) solution and images were captured with a luminescent image analyzer LAS-4000 mini (FUJIFILM, Tokyo, Japan). The quantification of band intensity on the blot was analyzed with a software program, Multi Gauge V3.0 (FUJIFILM, Tokyo, Japan).

### RNA interference experiment

Q7 and Q111 (1 × 10^5^ cells/ml) were transiently transfected with 100–400 nM of control short hairpin RNA (shRNA) and ESETshRNA using Lipofectamine™ 2000 transfection reagent (Invitrogen) in the presence or absence of HrD-IRES reporter for 48 h (30).

### Tet-inducible ESET (H3K9-specific histone methyltransferase) cell line

The T-RExTM System (Invitrogen) was used to generate ESET cell lines. This system utilized two vectors, the pcDNA6/TR vector, a regulatory plasmid that expresses the tetracycline repressor and pcDNA5/TO that contains a cytomegalovirus (CMV) promoter driving the expression of the gene of interest under the control of Tet-operator sequences. Myc-ESET was subcloned into the pcDNA5/TO vector from pcDNA-Myc-ESET construct, in which full length of ESET is cloned to a CMV-driven vector (Clontech). pcDNA5/TO-Myc-ESET was linearized and transfected into Q7 striatal cell clone over-expressing pcDNA6/TR. The ESET cell clones were selected by hygromycin. For the induction of ESET, 4 μg/ml of doxycyline was treated into culture medium.

### Run-on transcription assay and immunofluorescence

Nascent RNA was detected using a method previously described, with slight modifications (37). Briefly, cells were incubated with 5 mM 5-FU (Sigma Aldrich) for indicated time and fixed with 4% paraformaldehyde for 30 min at 4°C. Incorporated 5-FU was labeled with anti-BrdU antibody (Accurate Chemical) for overnight at 4°C and with Alexa Fluor 594-conjugated anti-mouse IgG antibody (Invitrogen) for 1 h. The nuclei were counterstained with DAPI, and labeled signals were visualized using fluorescent microscopy (Olympus). For the quantitative analysis, emitted signals for 5-FU was captured and their intensities were analyzed using multigauge program (Fuji).

### Chromatin preparation

Chromatin preparation was performed on the basis of described protocols (38,39). Tet-inducible ESET Cells were washed twice with ice-cold 1X PBS buffer supplemented with 0.1% Tween, then suspended in 500 μl of ice-cold lysis buffer (20 mM Tris pH8.0, 2 mM MgCl_2_). NP-40 (10 μl) was gently mixed in to release nuclei, and cells were immediately put back on ice for an additional 2 min. Released nuclei were spun down at 3000 rpm in 4°C for 10 min, rinsed once with fresh lysis buffer and resuspended in 0.1 M TE buffer (10 mM Tris, 1 mM EDTA, 100 mM NaCl) for subsequent Micrococcal nuclease (MNase) digestion. 0.5 U/ml MNase (Sigma Aldrich) was added for 0.5, 1, 2, 4 and 8 min at 37°C and 30 min at room temperature. MNase digests were stopped with 5 mM EDTA on ice. MNase-digested nuclei were spun down at 1500 rpm, resuspended in ice-cold nuclear extraction buffer (350 mM NaCl_2_, 2 mM EDTA, 0.5 mM PMSF in PBS) and left in an end-over-end rotary shaker at 4°C for 16 h. The supernatant (soluble chromatin extract) was used as input bulk chromatin for IP.

### IP for atomic force microscope

The 5 μg of anti-UBF (H-300, Santa Cruz Biotech.) was added in soluble chromatin extract (200 μg/ml) and incubated overnight at 4°C with rotation. The immune complexes were attached to 50 μl magnetic beads (Diagenode) and incubated for 2 h at 4°C with rotation. Elution was performed in 0.2 M Glycine in 1X PBS, pH6.5. Chromatin was dialyzed (Thermo Scientific) for 12 h against 1X PBS prior to imaging by atomic force microscope (AFM).

For AFM imaging, one drop of UBF IP samples were placed on freshly cleaved mica, incubated for 10 min at room temperature and then laid on the XE-100 AFM apparatus (Park Systems Inc.). The mica was performed using the non-contact mode.

### Statistical analysis

The data are presented as the mean ± SEM. Data analysis was performed by Student’s *t*-test using SigmaPlot 2000 (Systat Software). Differences were considered statistically significant when *p* < 0.05.

## RESULTS

### The level of ESET is increased in the nucleolus of HD cell lines

**Figure 1.**
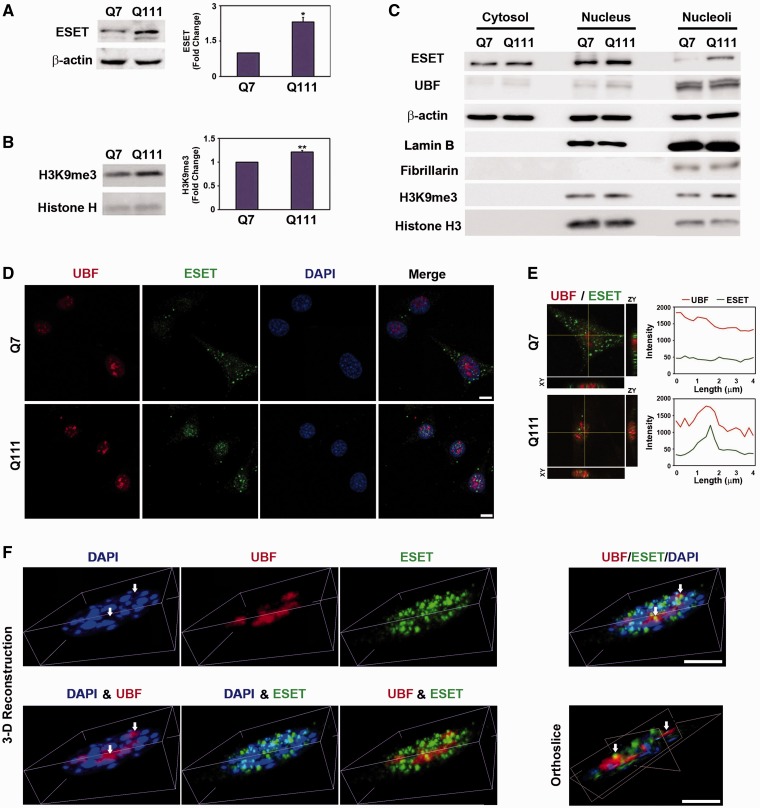
ESET is localized in the nucleolus of a cell line model of neurodegeneration. Increased levels of ESET (*n* = 5) (**A**) and H3K9me3 (*n* = 3) (**B**) were found in ST*Hdh*^Q111/111^ (Q111) cells in comparison with ST*Hdh*^Q7/7^ (Q7) cells. Significantly different from control at *P < 0.05; **P < 0.005. (**C**) The level of ESET was elevated in the nucleolar fraction in where UBF, a nucleolar transcription factor, is mainly found. The purity of subcellular fraction was determined by specific antibody as follows: Lamin B, a nucleus marker; Fibrillarin, a nucleolus marker. (**D**) The immunoreactivity of ESET was localized in the nucleolus of Q111 cells in comparison with Q7 cells. Scale bar: 10 µm. (**E**) The line measurement of ESET and UBF immunofluorescence signals showed their co-localization in the nucleolus of Q111 cells but not in Q7 cells. (**F**) Analysis of the co-localization of UBF with ESET using deconvoluted and 3D reconstructed confocal images. The 3D reconstructed orthoslice image confirms that ESET is co-localized in the peripheral regions of UBF foci in Q111 cells. White arrows indicate DAPI-free hollow regions (nucleolar compartments). Scale bar: 5 µm.

In the first series of experiments, we determined whether the level of ESET/SETDB1, a histone H3K9-specific methyltransferase and H3K9me3, a known ESET-specific substrate, are altered in a cell line model of HD. The protein levels of ESET and H3K9me3 were markedly increased in ST*Hdh^Q111/Q111^* (Q111) mutant cells in comparison with ST*Hdh^Q7/Q7^* (Q7) control cells as shown by western blot analysis (Figure 1A and B). The analysis of subcellular fraction showed that ESET and H3K9me3 were detected in nuclear and nucleolar fraction of Q111 cells (Figure 1C). We examined the purity of nuclear and nucleolar fraction using specific makers such as lamin B, a nucleus marker and fibrillarin, a nucleolus marker (Figure 1C). Consistent with subcellular fraction data, confocal microscopy showed that, in part, ESET is localized in the nucleolus of Q111 cells (Figure 1D). The punctate structure of ESET immunoreactivity was co-localized with UBF, a nucleolar transcription factor, in the nucleolus of Q111 cells (Figure 1E). In order to more clearly visualize the co-localization of UBF and ESET, 3D confocal images were reconstructed using the AQI-X-COMBO-CWF program (Media cybernetics Inc.). Figure 1F shows that UBF is mainly found in DAPI-free hollow regions (nucleolar compartments). The orthoslice view of the merged image clearly shows that ESET is co-localized in the peripheral region of UBF foci in Q111 cells (Figure 1F) as well as in Q7 cells (Supplementary Figure S2A). To provide more convincing data regarding the interaction and co-localization of UBF with ESET, we applied BiFC systems. BiFC is based on the formation of a fluorescent complex by two non-fluorescent fragments of Venus, VC155 and VN155, brought together by the association of proteins fused with each Venus fragment. In this context, UBF and ESET were fused with each Venus fragment and pBiFC-VC155-UBF and pBiFC-VN155-ESET were transfected to Q7 and Q111 cells. pBiFC-VC155 and pBiFC-VN155 was used as a negative control. As a result, the green fluorescence protein (GFP) fluorescence, a marker of UBF and ESET co-localization, appeared in the nucleolus of Q111 cells, while it was not detected in the nucleolus of Q7 cells. In contrast, pBiFC-VC155 and pBiFC-VN155 did not show GFP fluorescence at all. Consistent with IP and confocal microscopy data, these BiFC data confirm that UBF interacts with ESET and that they are co-localized in the nucleolus of Q111 cells (Supplementary Figure S1A). The co-localization of ESET and UBF was also found in HT22 (mouse hippocampal neuronal cell line) and SH-SY5Y (human neuroblastoma cell line) (Supplementary Figure S2B and C). Interestingly, the co-localization of ESET and UBF was increased in striatal neurons of HD transgenic (YAC+/−) mouse in comparison with control (YAC−/−) mouse in a similar manner to the cell line model of HD (Q7 versus Q111 cells) (Figure 1F; Supplementary Figure S2A and D).

### UBF interacts with ESET and its methylation is modulated by the methyltransferase activity of ESET

**Figure 2.**
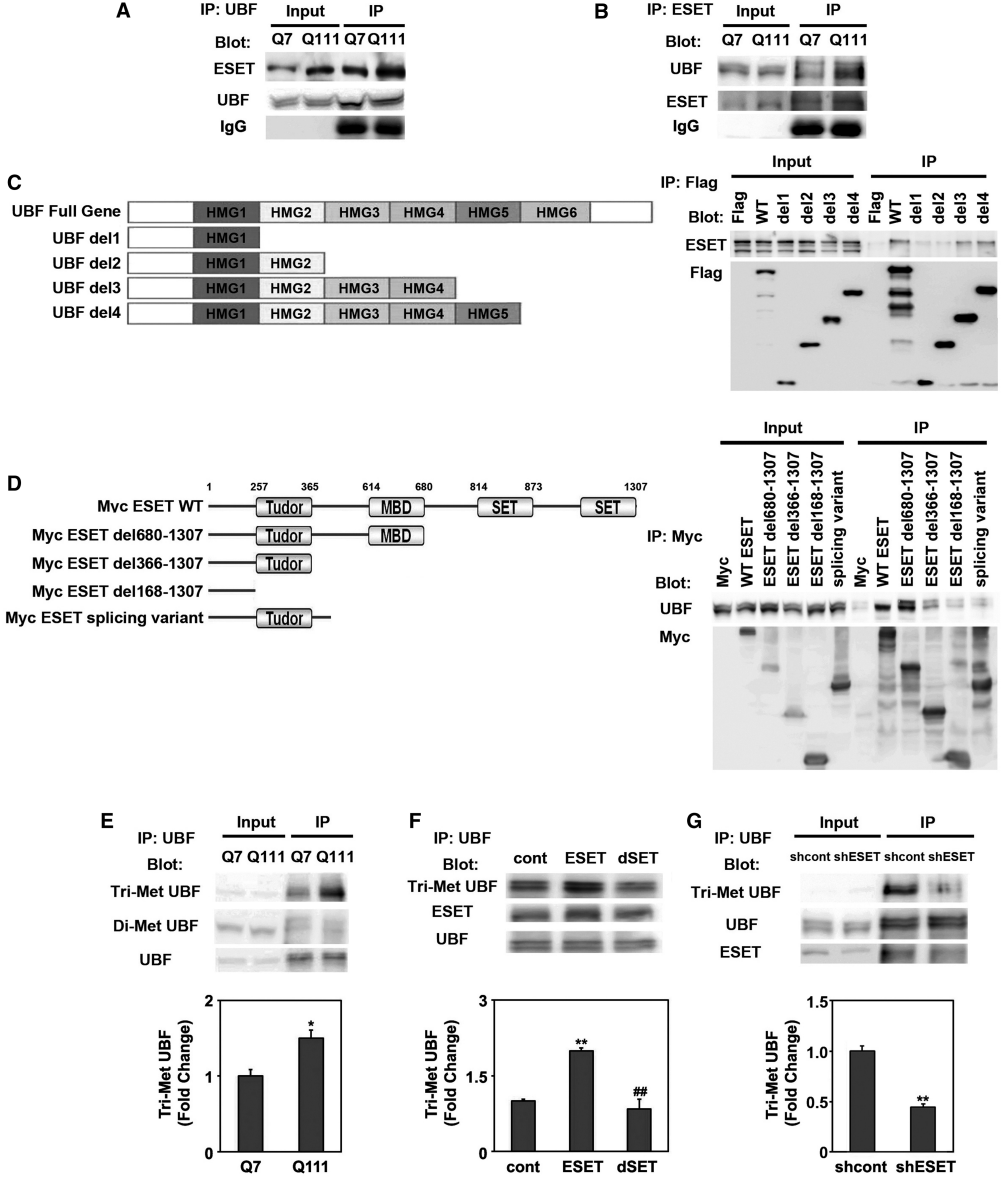
UBF interacts with ESET and its methylation is modulated by the methyltransferase activity of ESET. (**A**) UBF interacts with ESET in Q7 and Q111 cells. Cell lysates were immunoprecipitated with anti-UBF antibody and subsequently the blot was probed with anti-ESET antibody. The same blot was stripped and reprobed with anti-UBF antibody. (**B**) ESET was associated with UBF. (**C**) A scheme of UBF deletion constructs. Plasmids encoding Flag-UBF and its deletion mutants were transiently transfected in striatal (Q7) cells. The lysates were immunoprecipitated with anti-Flag antibody and the blot was probed with anti-ESET antibody. (**D**) Schematic representation of ESET functional domains. Plasmids encoding Myc-ESET and its mutants were transfected into Q7 cells. The lysates were immunoprecipitated with anti-Myc antibody and blots were probed with anti-UBF antibody. (**E**) The level of Tri-Met UBF was increased in Q111 HD cells in comparison with Q7 control cells. The UBF was immunoprecipitated and the blot was probed with anti-Tri-Met-Lys antibody (*n* = 3). (**F**) The deletion of SET domain in ESET (ESET–dSET) abrogated the methylation of UBF (*n* = 3). (**G**) Knockdown of ESET by shRNA reduced the methylation of UBF (*n* = 3). ESET (F) and UBF blots (G) were obtained from duplicate gels.

As we found the co-localization of ESET and UBF in the nucleolus, we characterized the *in vivo* association of UBF and ESET by performing IPs on neuronal lysates, using either anti-UBF or anti-ESET antibodies. The association of UBF and ESET by anti-UBF IP was apparent in both Q7 and Q111 cells (Figure 2A). Immunoblotting of anti-UBF IP samples with anti-UBF antibody verified that the same amounts of UBF were recovered by IP. We further confirmed the association of ESET and UBF using a reverse IP with ESET antibody (Figure 2B). Thus, our data confirmed that the UBF and ESET interact constitutively in intact cells. Next, in order to identify which region of UBF directly binds with ESET, we transfected Flag-tagged UBF deletion mutants in Q7 cells. The deletion of HMG2 and HMG3 domains reduced the binding of UBF to ESET (Figure 2C). Six separate GST–HMG domains (1–6) of UBF protein were purified and GST pull-down assays were performed with striatal cell lysates. Among six GST–HMG fusion proteins, the HMG2 and HMG3 domains of UBF interacted with ESET (Supplementary Figure S3A). To determine which domain of ESET interacts with UBF, we transfected Myc-tagged ESET deletion mutants in Q7 cells. Interestingly, strong UBF signals were detected on the Myc-ESET del680-1307, whereas a weak UBF signal was detected on the Myc-ESET del366-1307 and Myc-ESET del168-1307 mutants (Figure 2D). Our data show that domains of UBF such as HMG2 and HMG3 bind to the methyl-CpG binding domain (MBD) and tudor domain including N-terminus of ESET. Our data suggest that the HMG2 and HMG3 domains of UBF closely interact with the MBD domain of ESET leading to changes in the molecular structure of UBF and the subsequent methylation of HMG2 domains by ESET (Supplementary Figure S3B).

After we confirmed the interaction of UBF and ESET, we further addressed the following questions: first, is UBF methylated in intact cells? Second, if the methylated form of UBF is found, dose ESET act as a UBF methyltransferase and affect UBF-mediated rDNA transcription? Third, and most importantly, which residue of UBF is specifically methylated by ESET? To this end, we checked the methylation status of UBF in HD cells (Figure 2E). Interestingly, we measured methylated UBF from UBF IPs using anti-trimethylated (Tri-Met) lys and anti-dimethylated (Di-Met) lys antibodies, and found that the levels of Tri-Met UBF were increased in mutant Q111 cells compared with WT Q7 cells. Di-Met UBF levels were not changed (Figure 2E). Over-expression of ESET significantly augmented Tri-Met UBF levels but a dominant-negative mutant (ESET-dSET) lacking the methyltransferase (SET) domain did not (Figure 2F). In contrast, knockdown of ESET using shRNA ESET reduced Tri-Met UBF levels without affecting basal expression of UBF (Figure 2G). We then tested whether ESET is responsible for UBF-mediated transcription of rDNA. As we expected, ESET reduced UBF-mediated rDNA transcription and the deletion of ESET methyltransfease activity restored UBF-dependent rDNA transcriptional activity (Supplementary Figure S4A and B). Otherwise, we further addressed whether the decreased transcriptional activity of rDNA is affected by the decreased levels of UBF but not by the ESET activity. We performed experiments and determined how much ESET affects the expression of UBF mRNA and protein. We further quantified western blot (Supplementary Figure S4C) and qRT–PCR data (Supplementary Figure S4D). In addition, in order to distinguish the specific effect of ESET on the transcriptional regulation of rDNA versus the expression of UBF mRNA and protein, we analyzed Δ change of UBF protein, UBF mRNA and UBF-mediated rDNA transcriptional activity by ESET (Supplementary Figure S4E). As a result, we found that ESET significantly represses the UBF-mediated transcriptional activity of rDNA rather than affecting the expression level of UBF protein and UBF mRNA. These data strongly support that ESET modulates the UBF-mediated transcriptional activity of rDNA.

### UBF is methylated at the K232/K254 residue by ESET and UBF methylation site mutants decrease rDNA transcriptional activity

**Figure 3.**
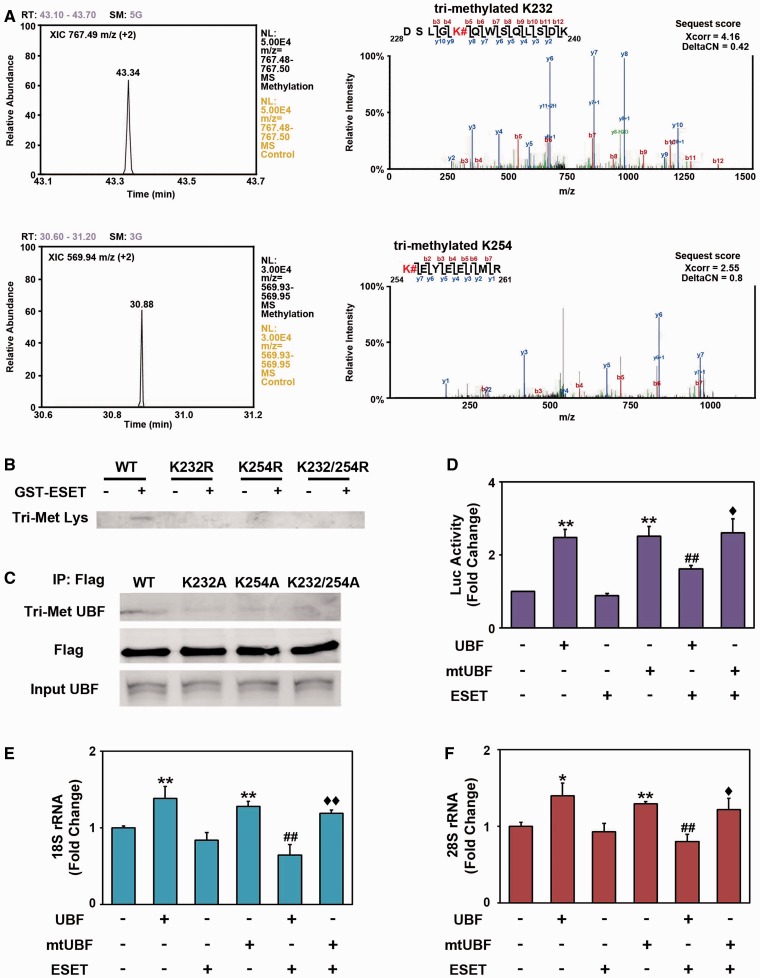
UBF is methylated at K232/K254 residues by ESET and the methylation status of UBF contributes to the transcriptional activity of rDNA. (**A**) Liquid chromatography-tandem mass spectrometry analysis identified the methylation site of UBF at K232 and K254 by ESET. The XIC of the trimethylated K232 peptide (DSLGK#QWSQLSDK, *m/z* = 767.49, doubly change) and tri-methylated K254 peptide (K#EYEEIMR, *m/z* = 569.94, doubly change) were extracted within the narrow m/z range (*m/z* = 767.48 to 767.50 in DSLGK#QWSQLSDK and *m/z* = 569.93 to 569.95 in K#EYEEIMR) using Xcalibur software (Thermo). Representative MS/MS spectra of trimethylated peptides (DSLGK#QWSQLSDK and K#EYEEIMR) are shown in right panels. Fragment ions detected in our study are labeled. The methylation site is marked with a sharp. The SEQUEST matching scores (Xcorr and deltaCN) of peptides were also shown. (**B**) Mutations at K232R, K254R and K232/254R of GST–UBF–HMG2 domain abrogated the trimethylation by ESET *in vitro*. (**C**) Ectopic expression of UBF methylation site mutants (K232A, K254A and K232/254A) reduced the methylation of UBF in intact cells. pCMV-Flag-UBFs (WT, K232A, K254A and K232/254A) were transiently transfected, immunoprecipitated with anti-Flag antibody and blotted with anti-Tri-Met-Lys antibody. The whole blots of Tri-Met Lys in (B) and Tri-Met UBF in (C) are presented in the Supplementary Figure S8. (**D**) The methylation site mutant of UBF (K232/254A double mutant, DM) resulted in the recovery of rDNA transcriptional activity in response to ESET (*n* = 5). The methylation site mutant UBF [mtUBF (K232/K254A)] restored 18S (**E**) (*n* = 5) and 28S (**F**) (*n* = 3) rRNA levels that were decreased by ESET. Double asterisk represents significantly different from control at *P* < 0.005; Hash represents significantly different from UBF at *P* < 0.05; Diamond represents Ssignificantly different from UBF with ESET at *P* < 0.05.

To identify which lysine (Lys) residue of UBF is directly methylated by ESET, we performed an *in vitro* methylation assay using purified GST–UBF–HMG1∼6 proteins and GST-ESET protein. As we found that GST–UBF–HMG2 and GST–UBF–HMG5 domains are specifically methylated by ESET (Supplementary Figure S4A), methylated GST–UBF–HMG2 and HMG5 domains were pooled and cut by PreScission Protease (GE Healthcare) for LC-MS/MS analysis. As shown in Figure 3, however, we identified that the Lys (K) 232 and 254 residues of HMG2 domain were specifically methylated by ESET (Figure 3A; Supplementary Figure S5B and C). We could not detect any methylated Lys residues in the HMG5 domain by LC-MS/MS. To further confirm whether UBF methylation at K232 and K254 are critical for transcriptional inhibition of rDNA, we generated K232 and K254 methylation site mutants using site-directed mutagenesis. We performed an *in vitro* methylation assay using UBF methylation site mutants (K232R, K254R and K232/254R) and GST-ESET protein. Methylation site mutants of UBF produced a marked reduction of ESET methylation (Figure 3B). As the mutation of one of the two target sites almost completely diminishes the methylation signal, we considered that the mutation of one site (K to A) among two target sites may change the molecular structure of UBF and subsequently prevent the methylation of non-mutated residue by ESET. In addition, as even one of methylation sites could affect the subtle stoichiometric change of molecular structure, the affinity of anti-trimethyl antibody may not be sufficient to clearly distinguish and detect the methylated signal in one of the two targets. Single mutations at K232A, K254A and double mutations at K232/254A reduced UBF methylation in normal striatal cells (Figure 3C) and in ESET inducible cells (Supplementary Figure S5D). These results directly confirm that UBF trimethylation at K232 and K254 by ESET occurs in intact neuronal cells. Mutation of UBF methylation sites (K232/254A: double mutant) restored the transcriptional activity of rDNA that was reduced by ESET, consistent with the reduction of Tri-Met UBF associated with mutations affecting methylation sites (Figure 3D). To determine the effects of ESET on the expression of rRNA, mature (18S and 28S) rRNA levels were measured by qRT–PCR. Consistent with the transcriptional activity of rDNA, the expression of 18S and 28S was decreased by ESET and its expression was restored by methylation site mutant UBF [mtUBF (K232/K254A)] (Figure 3E and F). The expression of intermediate (5′ETS) rRNA was also repressed by ESET and its expression was derepressed by mtUBF (K232/K254A) (Supplementary Figure S6A). Our qPCR data indicate that the transcriptional activity of UBF on 5′ETS is significantly increased but this increase is relatively small compared with levels of other rDNA transcripts such as 18S and 28S. The reason for this differential effect is unclear and further studies will be needed to clarify the mechanisms involved.

### ESET ON and OFF regulates the trimethylation of UBF and the transcription of rDNA

**Figure 4.**
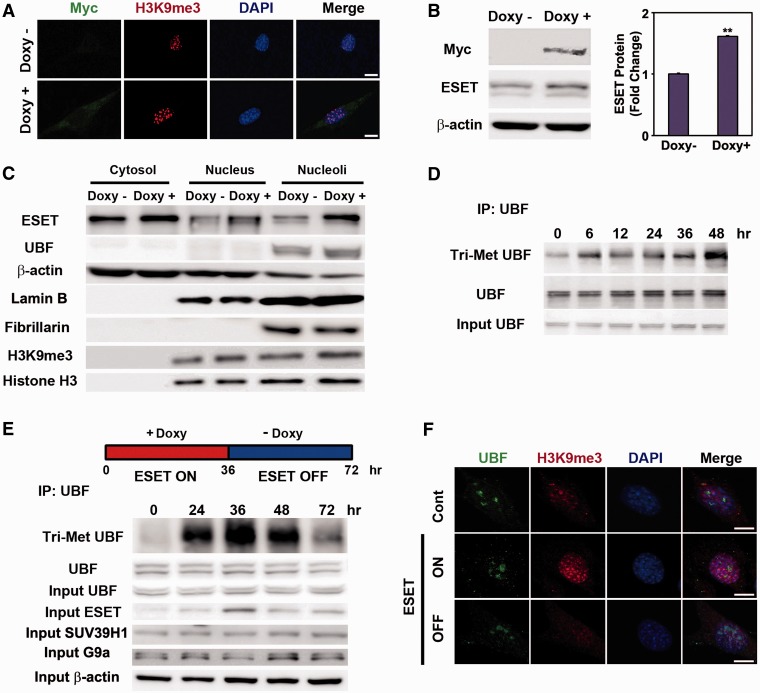
The level of trimethylated UBF (Tri-Met UBF) is regulated by ESET. (**A**) The immunoreactivity of Myc-tagged ESET and H3K9me3 was increased by doxycycline (Doxy). (**B**) The protein level of Myc-tagged ESET was robustly induced by Doxy (*n* = 3). (**C**) The increased levels of ESET and TMH3K9 were found in nuclear and nucleolar fractions in response to Doxy. The purity of subcellular fraction was determined by specific antibody as follows: Lamin B, a nucleus marker; Fibrillarin, a nucleolus marker. (**D**) The induction of ESET by doxycycline for the indicated period of time increased the expression trimethylated UBF in a time-dependent manner. (**E**) Cells were treated with doxycycline for 36 h (ESET ON) and then cells were washed of doxycycline and switched to the normal fresh media for 36 h (ESET OFF). The level of Tri-Met UBF was increased after 36 h of the ESET ON condition and was decreased to the basal levels after 36 h of the ESET OFF condition and vice versa. This change was depended on the induced or reduced of ESET. The whole blot of Tri-Met UBF in (E) is presented in the Supplementary Figure S8. (**F**) H3K9me3 immunoreactivity was markedly induced in ESET ON cells compared with ESET OFF cells. Scale bar: 10 µm.

To examine the role of ESET on UBF methylation and UBF-mediated nucleolar chromatin structure, we generated a Tet-inducible ESET cell line expressing Myc-tagged ESET. The addition of doxycycline to the medium resulted in the expression of Myc-tagged ESET and increased levels of H3K9me3. ESET protein was markedly increased by addition of doxycycline (Figure 4A and B). The ESET and H3K9me3 were increased in the nuclear and nucleolar fraction of doxycycline-treated cells (Figure 4C). Especially, the level of ESET was significantly increased in the nucleolar fraction (Supplementary Figure S7). The purity of nuclear and nucleolar fraction was confirmed using specific markers such as lamin B, a nucleus marker and fibrillarin, a nucleolus marker (Figure 4C). To test whether the methylation status of UBF is altered by ESET induction in the presence of doxycycline, we detected methylated UBF using anti-Tri-Met lys antibody on UBF IPs. As we expected, we found that the signal of Tri-Met UBF was evident at 6 h and increased up to 48 h in the presence of doxycycline (Figure 4D). After we validated the inducible ESET cell line, we established an ESET ON/OFF system. The Tet-inducible ESET cell line was treated with doxycycline for 36 h to turn the expression of ESET ON. Then, the cells were washed of doxycycline and switched to fresh normal media for 36 h to turn the expression of ESET OFF. The levels of Tri-Met UBF and ESET were significantly increased after 36 h of the ESET ON condition in comparison with control cells (0 h). The levels of Tri-Met UBF and ESET were decreased to the basal levels after 36 h of the ESET OFF condition (the total period of an ESET ON–OFF culture cycle was 72 h) (Figure 4E). Confocal microscopy confirmed that H3K9me3 immunoreactivity is increased in the ESET ON condition and it is reduced to basal levels in the ESET OFF condition (Figure 4F). Importantly, the expression of rRNA was down-regulated during the ESET ON condition and its expression was recovered during the ESET OFF condition (Supplementary Figure S6B). Levels of 18S and 28S RNA were reduced during the ESET ON condition in a manner similar to that seen in HD mice (Supplementary Figure S6C) and cell lines (Supplementary Figure S6D).

### UBF methylation by ESET affects its binding to rDNA and modulates nucleolar chromatin structure and plasticity

**Figure 5.**
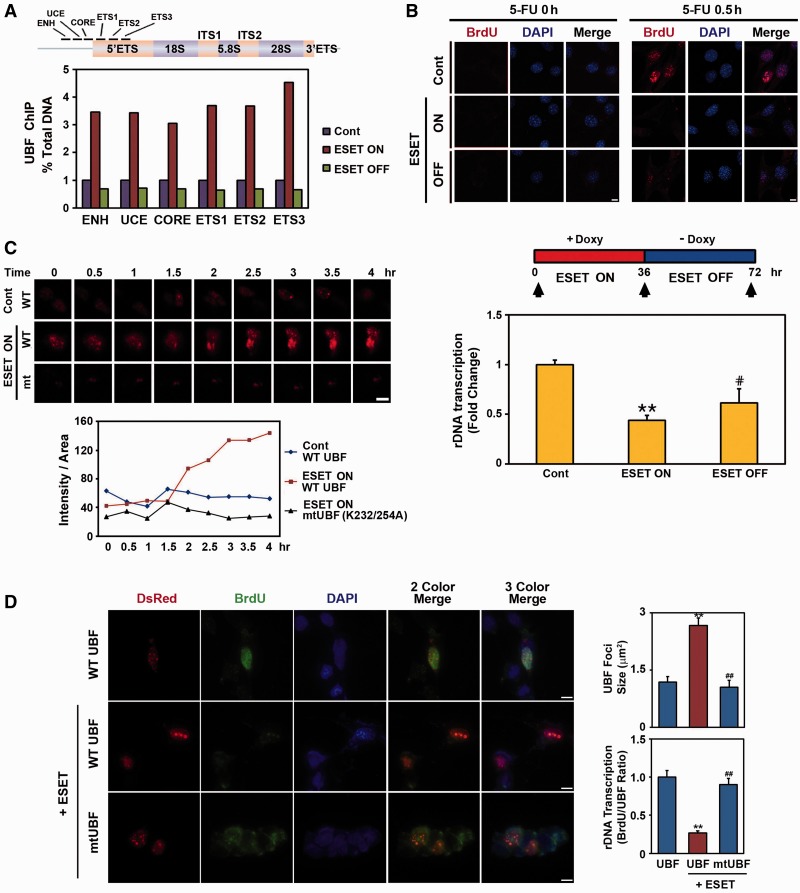
ESET modulates the occupancy of UBF to rDNA and the expression of rRNA. (**A**) Positions of qRT–PCR primer set for detecting UBF occupancy in the promoter of mouse rDNA (upper panel). Quantitative ChIP analysis of UBF occupancy in the mouse rRNA gene showed that the UBF binding to rDNA is dependent on ESET induction (bottom panel). (**B**) *In situ* transcription assay of 5′-FU incorporation into nucleolar rRNA showed the reduction of rRNA expression in ESET ON cells compared with control and ESET OFF cells (upper panel). The nucleus was counterstained with DAPI. The density (pixels) of BrdU immunoreactivity was averaged by counting each 60 cells in four areas. (**C**) Time-lapse microscopic analysis showed condensation of nucleolar chromatin in a UBF methylation-dependent manner during the ESET ON condition. (**D**) UBF methylation by ESET leads to reduced rRNA expression. Q7 cells were transiently transfected with UBF and mtUBF (K232/254A) or with ESET. UBF foci were increased in size by ESET (right upper panel). Mutant UBF methylation restored rDNA transcription compared with WT UBF (right bottom panel). The density of BrdU immunoreactivity was averaged by counting each 30 cells in 10 areas. Double asterisk represents significantly different from control cells at *P* < 0.005; hash represents significantly different from ESET ON cells at *P* < 0.05. Scale bar: 10 µm. ENH, enhancer; UCE, upstream control element; CORE, core region; ETS, external transcribed spacer.

To investigate how the methylation of UBF by ESET alters its occupancy of the rDNA promoter, we performed UBF ChIP and qRT–PCR assays under ESET ON and OFF conditions. Interestingly, the occupancy of UBF to UCE, CORE, ENH, ETS1, ETS2 and ETS3 regions of the rDNA promoter was markedly increased in ESET ON cells compared with control cells without ESET induction (Figure 5A). As we expected, the occupancy of UBF to UCE, CORE, ENH, ETS1, ETS2 and ETS3 regions were reduced to basal levels when cells returned to the ESET OFF condition. Next, to determine whether ribosomal biosynthesis is affected by UBF methylation produced by ESET, cells were pulse labeled for 30 min with 5-fluorouracil (FU) and immunolabeled with a mouse anti-BrdU antibody to visualize nascent ribosomal nucleolar transcripts. Incorporation of 5-FU was significantly decreased in ESET ON cells compared with ESET non-induced control cells (Figure 5B). The level of ribosome biosynthesis recovered in ESET OFF cells when doxycycline was washed out and replaced by fresh culture medium for 36 h (Figure 5B). Using time-lapse microscopy, we showed the nucleolar dynamics of UBF in ESET ON and OFF conditions. We transfected pHc-RED-WT UBF and pHc-RED-mutant UBF (K232/254 A) in controls and in ESET ON cells and imaged UBF trafficking for the indicated period of time. Marked UBF condensation occurred in the nucleolus after 2 h of doxycycline exposure (ESET ON), whereas the methylation site mutant of UBF (K232/254 A) did not show condensation of the fluorescence signal (Figure 5C). This result shows that the induction of ESET can directly drive the condensation of nucleolar chromatin structure through UBF methylation. To further examine the direct effect of ESET-dependent UBF methylation on rDNA transcription, we transiently transfected UBF and mtUBF (K232/254 A) together with ESET in Q7 cells and performed an *in situ* rDNA transcription assay by measuring 5′-FU incorporation into nucleolar rRNA. Consistent with data from Tet-on inducible ESET cell line (Figure 5B and C), co-transfection of WT UBF and ESET reduced rDNA transcription, while increasing the size of UBF foci (Figure 5D). In contrast, co-transfection of mtUBF (K232/254 A) and ESET restored rDNA transcription levels and maintained the size of UBF foci. These data suggest that the methylation of UBF by ESET directly involves rDNA transcription.

**Figure 6.**
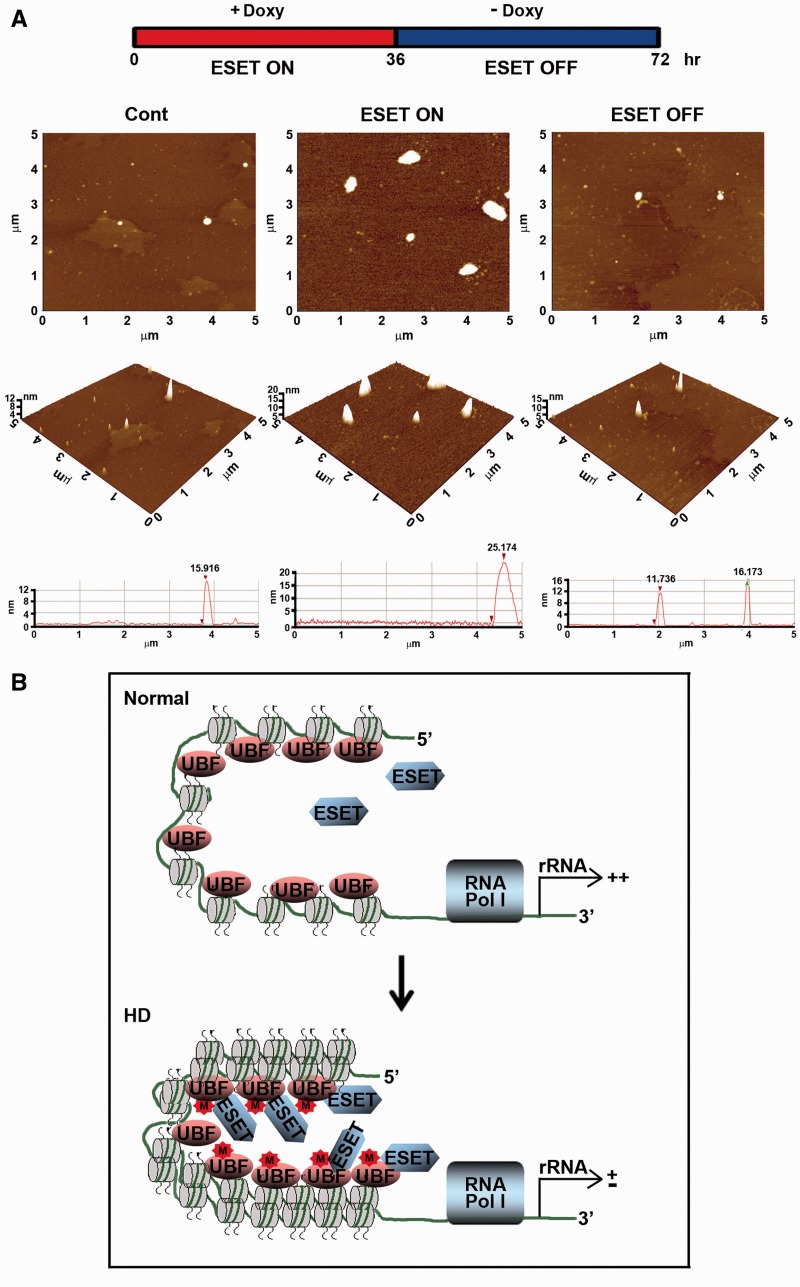
Methylation of UBF by ESET increases the condensation of UBF-mediated nucleosomal structure and the plasticity of chromatin in the nucleolus. (**A**) AFM analysis and representative 3D topography images showed a change in UBF-dependent nucleosomal structure in ESET ON cells compared with ESET OFF cells (middle panel). Single-molecule line scans showed that the height and distance of UBF-associated nucleosomes are robustly increased during the ESET ON condition (bottom panel). (**B**) A scheme illustrating that ESET modulates UBF-mediated chromatin remodeling and rDNA transcription in both the normal and the ESET ON conditions, similar to HD.

As we found that methylated UBF by ESET modulates rDNA transcription and nucleolar chromatin condensation at the cellular level, we proposed that the molecular level of chromatin remodeling might be correlated with the methylation status of UBF by ESET. Accordingly, in view of the direct role of ESET in influencing the structure of nucleolar chromatin through UBF methylation, we performed UBF IP with purified nucleosomes from intact cells under ESET ON and OFF conditions and examined nucleosomal structures using atomic force microscopy (AFM). Figure 6A showed UBF-immunoprecipitated histone complexes and a set of height measurements for the nucleosome complex. Consistent with the proposed hypothesis, the UBF-associated nucleosomal particles displayed increased heights and distances under the ESET ON condition compared with control cells without ESET induction. In contrast, the height and distance of UBF-associated nucleosomal particles was restored to their basal size in the ESET OFF cells (Figure 6A). These data indicated that both the induction level of ESET and the methylation status of UBF reversibly regulate the nucleolar chromatin condensation. As a result, in the context of epigenetic and mechanistic alterations of UBF methylation by ESET, we suggest a scheme showing that ESET modulates UBF-mediated chromatin remodeling and rDNA transcription (Figure 6B). In normal conditions, the lower level of ESET may not hinder the UBF-mediated nucleolar chromatin remodeling and rDNA transcription, but under HD condition, the increased ESET and the subsequent elevation of UBF methylation involves in the condensation of nucleolar chromatin and leads to the deregulation of rDNA transcription (Figure 6B).

## DISCUSSION

Dynamic nucleolar chromatin remodeling regulates discrete molecular interactions and governs rDNA transcription and ribosome biogenesis (2). However, the extent to which structural nucleolar proteins contribute to this level of organization is largely unresolved. To test the links between structure and function, we evaluated how ESET and UBF contribute to the chromatin organization of the nucleolus. We recently reported that UBF-mediated rDNA transcription is activated by acetyltransferase activity of CBP (19). We further determined that neurodegenerative stress associated with HD can change adaptive rDNA transcription by altering the acetyltransferase activity of CBP and affecting the balance of acetylated and deacetylated UBF (13). Although the acetylation of UBF supports robust rDNA gene transcription, its relationship to post-translational modifications such as methylation of UBF is not known.

In this study, we found that increased levels of methylated UBF are correlated with the increased ESET activity, a pathological event that is found in HD. Our data show that ESET physically interacts with UBF and methylates it in intact neurons. The methyltransferase activity of ESET is directly responsible for UBF methylation. Accordingly, SET domain deletion mutants of ESET resulted in a marked reduction of UBF methylation. In general, K (lysine) residues are mono-, di- or trimethylated and the status of methylation contributes to different functional outcomes (40). We confirmed that UBF is presented as a trimethylated form in intact cells. In addition, UBF is trimethylated by ESET *in vitro*. To identify the specific methylation site of UBF by ESET, we performed LC-MALDI-MS/MS analysis *in vitro* on UBF-HMG protein methylated by ESET. We found that ESET methylates UBF at the K232/254 residues of the HMG2 domain. Accordingly, mutations at K232/254 to alanine (A), glutamine (Q) or arginine (R) blocked UBF trimethylation and derepressed the transcriptional activation of UBF by ESET. As expected, consistent with the transcriptional activity of rDNA, methylation site mutant UBF (K232/254 A) restored the transcriptional level of intermediate (5′-ETS) and mature (18S and 28S) rRNA levels while ESET decreased the expression of rRNA through wild-type UBF.

HD is caused by an expansion of CAG repeats coding for glutamine (Q) in exon 1 of the *Htt* gene. The motor symptoms of HD are largely a consequence of profound neurodegeneration in the striatum (41). Deregulation of chromatin remodeling is regarded as one of the mechanisms by which mutant Htt (mtHtt) contributes to neurodegeneration (20). MtHtt not only blocks the intrinsic HAT activity of CBP but also induces epigenetic enzymes (14,31). These specific epigenetic alterations show how mtHtt modulates transcriptional signaling cascades that initiate a number of downstream pathophysiological mechanisms relevant to HD. As we expected, we found that levels of ESET and methylated UBF in the striatum were significantly increased in R6/2 transgenic HD mice and in human HD tissue. We also determined that 45S levels were down-regulated by ESET. Our data suggest that the increased level of methylated UBF is correlated with deregulation of ribosomal transcription in HD*.*

In order to address whether epigenetic modification of UBF by ESET is reversibly regulated *in vivo*, we generated a Tet-on ESET inducible cell line. Although the UBF-mediated nucleolar nucleosomal structure and chromatin condensation were increased by ESET induction, nucleosomal structure and chromatin condensation were decreased to basal levels by turning off ESET induction. Thus, our data prove that the ESET-dependent trimethylation of UBF plays a direct role in the regulation of nucleolar chromatin plasticity. Targeting of UBF trimethylation to regions of heterochromatin is sufficient to induce large-scale chromatin condensation in the nucleolus. In this regard, the binding of trimethylated UBF throughout the rDNA gene repeat might contribute to the formation of the inactive chromatin state of rDNA genes. The novel role of UBF trimethylation produced by ESET in maintaining nucleolar chromatin plasticity suggests that a steric non-histone molecule such as UBF is essential to landscape the chromatin structure of the nucleolar compartment (42). Moreover, our data indicates that ESET functions as a regulator of both nuclear and nucleolar chromatin remodeling. It remains to be determined, however, how mtHtt and HD-related cellular changes modulate the association of ESET to the UBF complex. It will also be important to precisely define the demethylation mechanism of UBF in order to more fully understand a dynamic role of UBF on nucleolar chromatin remodeling and rDNA transcription in HD.

Taken together, our findings show that trimethylated UBF at K232/254 by ESET leads to the condensation of nucleolar chromatin and deregulates the transcriptional activity of rDNA. The deregulation of rRNA expression by trimethylated UBF contributes to the molecular pathology of HD. Nucleolar chromatin is highly plastic with inducible ESET expression and its reversible structural changes are correlated with the demand for rDNA transcription. Our data suggest that ESET and trimethylated UBF-mediated chromatin remodeling improves our understanding of neuronal rDNA transcription and its role as an epigenetic mechanism relevant to neurodegenerative conditions.

## SUPPLEMENTARY DATA

Supplementary Data are available at NAR Online.
